# Bone sarcoma follow-up; a nationwide analysis of oncological events after initial treatment

**DOI:** 10.1016/j.jbo.2022.100466

**Published:** 2022-12-09

**Authors:** Louren M. Goedhart, Vincent K.Y. Ho, Joris J.W. Ploegmakers, Ingrid C.M. van der Geest, Michiel A.J. van de Sande, Jos A. Bramer, Martin Stevens, Paul C. Jutte

**Affiliations:** a Department of Orthopaedics, University of Groningen, University Medical Center Groningen, The Netherlands; b Netherlands Comprehensive Cancer Organisation (IKNL), Utrecht, The Netherlands; c Department of Orthopaedics, Radboud University Medical Center, Nijmegen, The Netherlands; d Department of Orthopaedics, Leiden University Medical Center, The Netherlands; e Department of Orthopaedics, Amsterdam University Medical Centers, Amsterdam, The Netherlands

**Keywords:** DFI, Disease-Free Interval, NCR, Netherlands Cancer Registry, PALGA, Dutch Pathology Network, ACT, Atypical Cartilaginous Tumour, NOS, Not Otherwise Specified, Chondrosarcoma, Osteosarcoma, Ewing sarcoma, Follow-up

## Abstract

**Aim:**

Follow-up strategies for high-grade bone sarcomas have been optimized to facilitate early detection of local recurrence and distant metastasis. The ideology is that early detection enables early treatment presuming better survival. However, the clinical value for each individual patient remains questionable. This study aims to evaluate oncological events after initial treatment in order to assess current follow-up strategies for high-grade bone sarcomas in the Netherlands.

**Patients and Methods:**

A retrospective cohort study was conducted based on a national registry. All cases were retrieved from the Netherlands Cancer Registry. Our study consisted of 393 patients treated between 2007 and 2011 with complete follow-up data. Baseline characteristics were analysed for all entities. Local recurrence and distant metastasis was analysed along with overall survival for high-grade chondrosarcoma, high-grade osteosarcoma, Ewing sarcoma and chordoma.

**Results:**

Median follow-up was 8,3 years for high-grade chondrosarcoma, 4,9 for high-grade osteosarcoma, 3,8 for Ewing sarcoma and 7,5 for chordoma. Median time to local recurrence and distant metastasis was 1,2 years for high-grade osteosarcoma and 1,5 years for Ewing sarcoma. For high-grade osteosarcoma with localized disease at presentation, 0.09 new distant metastatic events per patient per year were seen after five years of follow-up with 11,1 patients needed to follow-up for any event. Five-year overall survival was 60,0% for high-grade chondrosarcoma, 50,0% for high-grade osteosarcoma, 45,3% for Ewing sarcoma and 71,4% for chordoma.

**Conclusions:**

This nationwide study shows a plateau in local recurrences and distant metastatic events after four years of treatment for patients with high-grade osteosarcoma and Ewing sarcoma. Due to a lack of reliable evidence however, we were not able to provide additional guidance on follow-up intervals and duration. Collaborative research with larger groups is needed in order to provide a solid scientific recommendation for follow-up in the heterogenous patient population with bone sarcoma.

## Highlights

• A plateau in new local recurrences and distant metastatic events four years after initial treatment was seen for patients with high-grade osteosarcoma and Ewing sarcoma.
• Collaborative research with larger groups is needed in order to do provide a solid scientific basis for future recommendations for follow-up interval and duration.
• The discussion regarding the purpose of extended follow-up and its value for the individual patient initially treated with curative intend should be intensified.

## 1 Introduction

For more than three decades, survival of high-grade bone sarcoma patients has not significantly improved despite multimodal treatment by experienced teams [1], [2], [3].

Recurrent disease (local or metastatic) is a strong negative prognostic factor for chondrosarcoma, osteosarcoma and Ewing sarcoma [4], [5], [6], [7], [8]. Follow-up surveillance programmes are aimed at early detection of local recurrence or metastatic disease presuming better survival with early detection. The follow-up concept is based on the hypothesis that early detection of recurrences would lead to smaller lesions that are more likely to be treated with success and less morbidity. Nevertheless, the efficiency and yield of radiologic detection of local recurrences using protocolised follow-up may be limited [9]. Pulmonary metastases are most frequently detected within the first two years of follow-up [10]. Furthermore, only a small subgroup of patients with metastatic disease is found eligible for additional treatment [11]. Promising results from pulmonary metastectomy have been published for osteosarcoma and Ewing sarcoma. A relatively long disease-free interval (DFI) between primary disease and metastatic disease was identified as an important beneficial prognostic factor [12], [13], [14]. The relatively low yield of protocolled radiographic follow-up along with low survival rates after treatment of metastasized bone tumours, questions the added value of follow-up for bone sarcoma patients treated with curative intend. Additionally, follow-up is time consuming, strains health care expenses and repeated CT imaging has late stochastic effects [15], [16]. Lastly, psychological distress during follow-up has been reported in up to 25 % of sarcoma patients [17].

Few studies regarding follow-up strategies and the effects on survival are known. Puri et al. found that a less intensive follow-up scheme was non-inferior in terms of recurrence free survival and overall survival [18]. Furthermore, a recent American/Canadian study based on a retrospective cohort proposed a follow-up protocol for high-grade bone sarcomas with prolonged intervals two years after treatment [10]. Consensus regarding follow-up in the existing guidelines is brief and only reinforced by low-level evidence, which questions the effectiveness of follow-up on survival for the individual patient [19].

Therefore, this nationwide study aims to evaluate the oncological events occurring after index treatment with curative intent during follow-up, including time to local recurrence and distant metastasis, in order to obtain additional evidence to assess current follow-up strategies for high-grade bone sarcomas in the Netherlands.

## 2 Material and Methods

A retrospective cohort study was conducted based on a national registry. All cases were retrieved from the Netherlands Cancer Registry (NCR), which receives primary notification from the Dutch Pathology Network (PALGA). This notification resulted in a complete and pathology based cohort. Patients who were treated between 2007 and 2011 were included in order to achieve a substantial follow-up period. Additional clinical information (on patient and tumour characteristics and treatment regimens) was collected by data managers of the NCR from hospitals’ patient records. Unlike most cancer registries, the NCR has no access to death certificates, which impedes reporting on the proportion of Death Certificate Initiated as well as Death Certificate Only cases. The Institutional Review Board of University Medical Center Groningen approved this study (M19.224412) and waived patient informed consent.

**Fig. 1:**
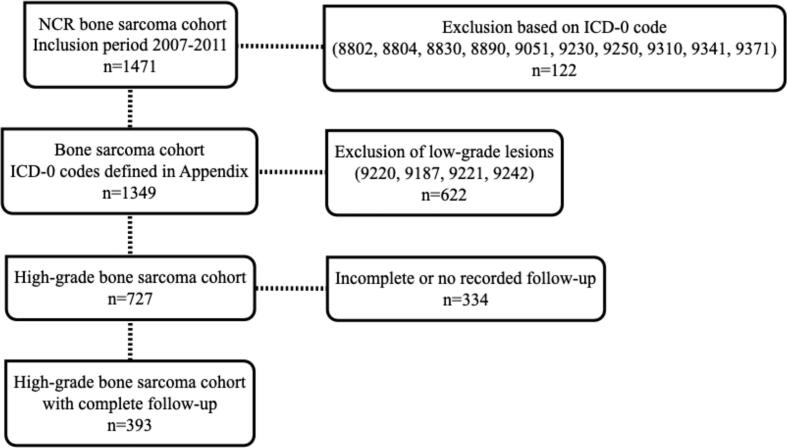
Flowchart for inclusion.

For inclusion, classification and categorisation of sarcoma in terms of localisation and histology were based on the International Classification of Diseases for Oncology (ICD-O-3) and the WHO classification 2013 applied according to our clinicopathological expertise as shown in Appendix 1. We excluded several entities from the original cohort, as shown in Appendix 1 as well. Additionally, we excluded patients with low-grade tumours as well as patients with incomplete follow-up in order to obtain a high-grade bone sarcoma cohort with complete follow-up. The flowchart for inclusion is shown in Fig. 1.

For analysis, descriptive statistics were used for the clinicopathological characteristics.

Time to local recurrence and distant metastasis along with metastatic events were calculated for high-grade chondrosarcoma, high-grade osteosarcoma, Ewing sarcoma and Chordoma. For the visualisation of the timing of local recurrence and distant metastasis, we used Kaplan Meier curves. For analysis of local recurrence and distant metastasis free survival, we performed a competing risk analysis Stata (Version 17.0; StataCorp, College Station, TX, USA). New distant metastatic events for patients with localized disease during follow-up were calculated using Poisson regression analysis in order to estimate incidence over time. Univariable overall survival analysis was performed for high-grade chondrosarcoma, high-grade osteosarcoma, Ewing sarcoma and chordoma using normal Kaplan-Meier curves. For survival analyses, information on patients’ vital status was obtained through linkage with the Municipal Personal Records Database. Statistical analysis was performed using SPSS software (Version 23.0; SPSS Inc, Chicago, IL, USA).

## 3 Results

### 3.1 Clinicopathological characteristics

A total of 393 bone sarcoma patients with complete follow-up, median age of 39 years, were included. High-grade chondrosarcoma was diagnosed in 104 patients, 144 patients with high-grade osteosarcoma were seen along with 55 Ewing sarcoma patients. Chordoma (n = 44), Surface osteosarcoma (n = 12), classic adamantinoma (n = 15), angiosarcoma of bone (n = 4) and sarcoma of bone Not Otherwise Specified (NOS) (n = 15) were also included.

Clinicopathological characteristics for high-grade chondrosarcoma, high-grade osteosarcoma, Ewing sarcoma and chordoma are displayed in Table 1. For the other sarcoma subtypes with lower incidences, these characteristics are shown in Table 2.

**Table 1:** Clinicopathological characteristics for high-grade chondrosarcoma, high-grade osteosarcoma, Ewing sarcoma and chordoma.

	High-grade chondrosarcoma (grade 2 / 3 / ddif) n = 104	High-grade osteosarcoma n = 144	Ewing sarcoma n = 55	Chordoma n = 44
Gender (%)	M 62 (59.6)F 42 (40.4)	M 81 (56.3)F 63 (43.8)	M 40 (72.7)F 15 (27.3)	M 30 (68.2)F 14 (31.8)
Median age at diagnosis in years (range)	55 (19–88)	22,5 (5–83)	19 (6–62)	63,5 (29–85)
Localisation (%)	Long bones 60 (57.7)Axial skeleton 44 (42.3)	Long bones 124 (86.1)Axial skeleton 20 (13.9)	Long bones 25 (45.5)Axial skeleton 30 (54.5)	Long bones -Axial skeleton 44 (100)
Extent of disease at time of diagnosis (%)	Localized 85 (81.7)Metastasized 19 (18.3)	Localized 107 (74.3)Metastasized 37 (25.7)	Localized 29 (52.7)Metastasized 26 (47.3)	Localized 27 (61.4)Metastasized 3 (6.8) Missing 14 (31.8)
Median follow-up in years (range)	8.3 (0–14)	4.9 (0.3–14)	3.8 (0.5–13.8)	7.5 (0.9–13.5)
Median time to Local Recurrence in years (range)	1.9 (0–10.7)	1.2 (0.2–13.25)	1.5 (0.5–12.7)	2.7 (0.7–6.9)
Median time to Distant Metastasis in years (range)	2.1 (0–10.7)	1.2 (0.2–13.25)	1.5 (0.5–12.7)	2.9 (0.7–7.3)

M = male; F = female; ddif = dedifferentiated; n = number.

**Table 2:** Clinicopathological characteristics for other bone sarcomas.

	Surface osteosarcoma n = 12	Adamantinoma n = 15	Angiosarcoma of bone n = 4	Sarcoma of bone NOS n = 15
Gender (%)	M 5 (41.7)F 7 (58.3)	M 8 (53.3)F 7 (46.7)	M 3 (75.0)F 1 (25.0)	M 6 (40.0)F 9 (60.0)
Median age at diagnosis (range)	29 (13–58)	14 (1–63)	63 (39–74)	52 (9–83)
Localisation (%)	Long bones 12 (100) Axial skeleton -	Long bones 15 (100) Axial skeleton -	Long bones 4 (100) Axial skeleton -	Long bones 8 (53.3)Axial skeleton 7 (46.7)
Disease extent at presentation (%)	Localized 4 (33.3)Metastasized 1 (8.3) Missing 7 (58.3)	Localized 15 (86.7)Metastasized 2 (13.3)	Localized 1 (25.0)Metastasized 3 (75.0)	Localized 10 (66.7)Metastasized 5 (33.3)
Median follow-up in years (range)	10.8 (5.2–13.7)	11.8 (7.3–13.9)	1.3 (0.3–13.3)	2.3 (0.1–13.3)

M = male; F = female;

### 3.2 Follow-up

Follow-up time, time to local recurrence and distant metastasis and overall survival was only analyzed for high-grade chondrosarcoma, high-grade osteosarcoma, Ewing sarcoma and Chordoma.

Median follow-up was 8.3 years for high-grade chondrosarcoma, 4.9 for high-grade osteosarcoma, 3.8 for Ewing sarcoma and 7.5 for chordoma. For these entities, time to local recurrence and distant metastasis in years for patients with localized disease are displayed in Table 1.

### 3.3 Local recurrence and distant metastasis

Five-year local recurrence rates for patients with localized disease was 37.6 % for high-grade chondrosarcoma, 21.5 % for osteosarcoma, 31.0 % for Ewing sarcoma and 51.9 % for Chordoma patients respectively. Five-year distant metastasis rates were 22.3 % for high-grade chondrosarcoma, 48.6 % for osteosarcoma, 55.1 % for Ewing sarcoma and 18.5 % for Chordoma. The incidence of distant metastasis during follow-up is defined as new distant metastatic events per patient per year for patients with localized disease at diagnosis. This is displayed in Table 3. Median time to local recurrence and distant metastasis is displayed in Table 1. The trends in timing of local recurrence and distant metastasis are visualised in Fig. 2a, Fig. 2b. For high-grade chondrosarcoma, a decrease in local recurrences and distant metastatic events occurred after approximately-seven years. For patients with high-grade osteosarcoma and Ewing sarcoma, a plateau in local recurrences and distant metastatic events was reached after approximately-four years. A different pattern was seen for patients with chordoma with ongoing events of local recurrence and metastasis during ten-year follow-up.. Competing risk analysis of local recurrence and distant metastasis free survival was displayed in Fig. 3a, Fig. 3b. For local recurrence free survival, correction for competing risks resulted in slightly better local recurrence free survival for all entities. For distant metastasis, no significant differences were seen.

**Table 3:** New Distant Metastatic events for patients with localized disease at diagnosis.

Entity	Follow-up in years	New Distant Metastasis per patient per year	Number of patients needed to follow-up* (CI)
High-grade chondrosarcoma n = 85 (95 % CI)	0–2	0.13 (0.08 – 0.21)	7.7 (4.8 – 12.5)
	2–5	0.03 (0.01 – 0.09)	33.3 (11.1–100)
	5–10	0.09 (0.04 – 0.20)	11.1 (5–25)
	>10	0	
High-grade osteosarcoma n = 107 (95 % CI)	0–2	0.30 (0.22 – 0.41)	3.3 (2.4 – 4.5)
	2–5	0.22 (0.12 – 0.38)	4.6 (2.6 – 8.3)
	5–10	0.09 (0.03 – 0.24)	11.1 (4.2 – 33.3)
	>10	0	–
Ewing sarcoma n = 29 (95 % CI)	0–2	0.24 (0.13 – 0.44)	4.2 (2.3 – 7.7)
	2–5	0.38 (0.17 – 0.83)	2.6 (1.2 – 5.9)
	5–10	0	
	>10	0	
Chordoma n = 27 (95 % CI)	0–2	0.02 (0.003 – 0.15)	50 (6.7 – 333.3)
	2–5	0.11 (0.04 – 0.30)	9.1 (3.3 – 25)
	5–10	0.37 (0.14 – 0.99)	2.6 (1.0 – 7.1)
	>10	No follow-up	–

*Number of patients needed to screen to detect 1 new metastatic event per year.

CI Confidence interval.

**Fig. 2a:**
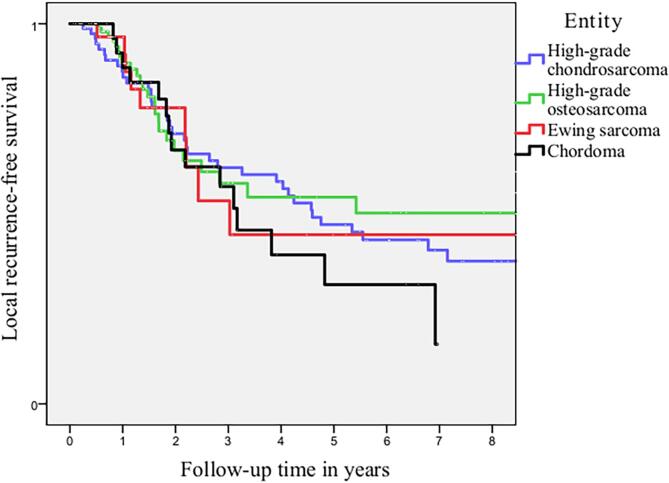
Time to Local Recurrence in localized disease for high-grade chondrosarcoma, high-grade osteosarcoma, Ewing sarcoma and Chordoma.

**Fig. 2b:**
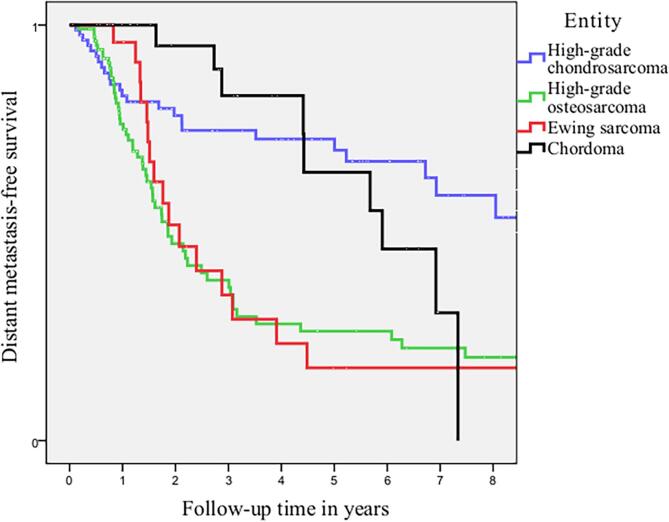
Time to Distant Metastasis in localized disease for high-grade chondrosarcoma, high-grade osteosarcoma, Ewing sarcoma and Chordoma.

**Fig. 3a:**
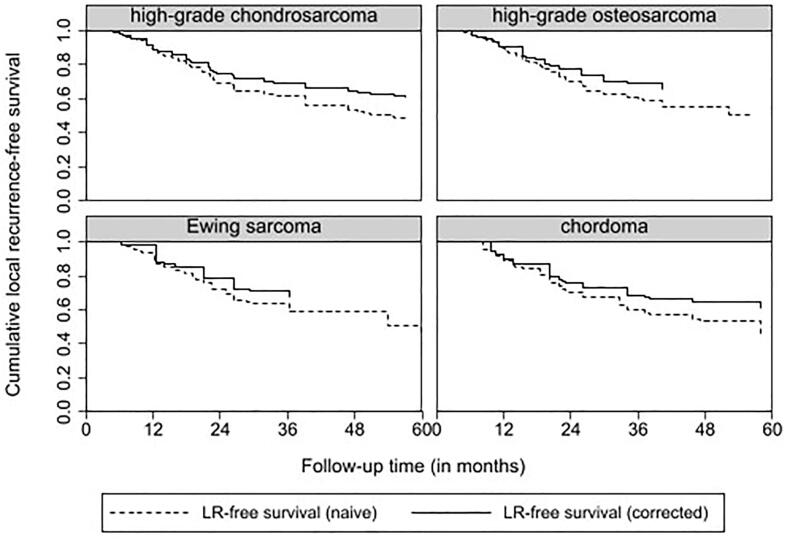
Competing risk analysis for local recurrence free survival for high-grade chondrosarcoma, high-grade osteosarcoma, Ewing sarcoma and chordoma.

**Fig. 3b:**
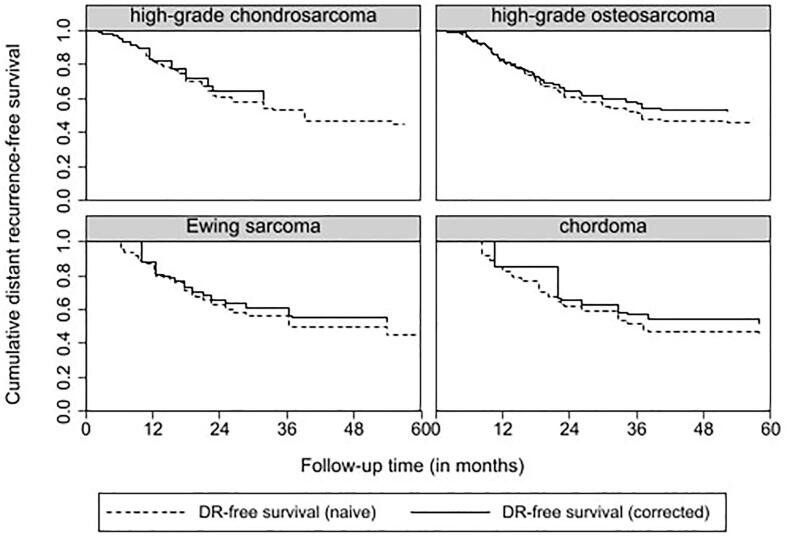
Competing risk analysis for distant metastasis free survival for high-grade chondrosarcoma, high-grade osteosarcoma, Ewing sarcoma and chordoma.

### 3.4 Survival

Median survival time in years, 2- and 5-year survival rates are displayed in Table 4 for patients with localized disease and the overall population. Five-year overall survival was 60.0 % for high-grade chondrosarcoma, 50.0 % for high-grade osteosarcoma, 45.3 % for Ewing sarcoma and 71.4 % for chordoma.

**Table 4:** Median, 2- and 5-year survival for high-grade chondrosarcoma, high-grade osteosarcoma, Ewing sarcoma and chordoma.

	N	median survival time in years (CI)	2-year survival (%)	5-year survival (%)
Chondrosarcoma (high-grade, grade 2/3/ddif)	100	8.3 (-)	78.0	60.0
High-grade osteosarcoma	140	4.7 (1.4–7.9)	65.7	50.0
Ewing sarcoma	53	3.8 (1.0–6.6)	66.0	45.3
Chordoma	42	7.3 (5.4–9.2)	92.9	71.4

CI Confidence Interval.

**Fig. 4:**
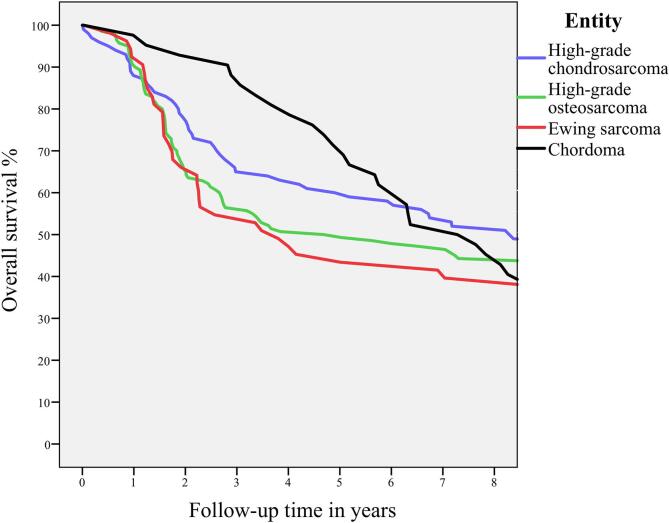
Overall survival for high-grade chondrosarcoma, high-grade osteosarcoma, Ewing sarcoma and chordoma.

Five-year overall survival for these entities is illustrated in Fig. 4. Survival more or less reaches a plateau after approximately-six years of follow-up as a result of the timing of appearance of local recurrence and metastasis for patients with high-grade osteosarcoma and Ewing sarcoma. This plateau in survival is reached after approximately-eight years for high-grade chondrosarcoma patients. Interestingly, the survival curve for chordoma is clearly different with a steady decline in survival during ten-year follow-up.

## 4 Discussion

With comprehensive evaluation of oncological events after treatment, we aimed to assess current follow-up strategies for high-grade bone sarcomas in the Netherlands. The NCR is a population-based registry that covers the total population of the Netherlands since 1989 (approximately 17,5 million inhabitants in 2022) [20]. At present, about 96 % of records concerns histologically verified cases, with the majority of remaining cases representing clinical diagnoses [21]. Only high-grade bone sarcomas were included in this study. As a result of extensive centralisation in the Netherlands it can be hypothesized that virtually all high-grade lesions are histologically verified with tumour biopsy [22]. Therefore, the rate of missing data in our study can be considered negligible. The results of our nationwide retrospective cohort study showed clear patterns for the occurence of local recurrence and metastasis during follow-up for high-grade bone sarcomas.

High-grade chondrosarcoma, high-grade osteosarcoma, Ewing sarcoma and chordoma were deemed eligible for the analysis of local recurrence, distant metastasis based on the sample size.

In Fig. 2, we used Kaplan Meier curves for visualisation of the trends in timing of oncological events after initial treatment. Due to competing risks and censoring, the Kaplan Meier curves in Fig. 2 do not resemble actual survival. The primary goal of these figures was to observe the trends in timing of local recurrence and distant metastasis, not to determine specific survival. For high-grade chondrosarcoma patients, on-going local recurrences and distant metastasis after seven years of follow-up were seen without a decrease in survival between seven and ten years of follow-up. However, it is uncertain if causality can be assumed with a median follow-up of 8,3 years. By performing a competing risk analysis as shown in Fig. 3 for local recurrence free survival, more accurate and slightly better local recurrence free survival was seen compared to survival analysis with Kaplan Meier curves.

For high-grade osteosarcoma patients, survival plateaus after approximately-six years of follow-up as a result of a stabilisation in the occurrence of local recurrence and distant metastasis after approximately-four years. Extended follow-up beyond five years seems of limited added value. However, with only a few patients left in follow-up after five years, a recommendation for extended follow-up is not justified.

A small increase in incidence of new distant metastatic events for patients with localized Ewing sarcoma was seen between two and five years of follow-up. However, a plateau in the timing of local recurrence and distant metastasis was reached after approximately-four years. A plateau in survival is reached after approximately-six years of follow-up, but this finding is doubtful as well with only a few patients left in follow-up.

Comparison of new distant metastatic events for patients with localized disease with existing literature proved to be difficult. Cipriano et al. defined their groups on gradation rather than entity and did not define extent of disease [10]. This gives a different, but still a valuable picture compared to the calculations in our study which was focused on patients with high-grade bone sarcomas with localized disease. In a comparable series from a single centre, most local recurrences and metastatic events for high-grade extremity osteosarcoma were seen within the first two years of follow-up [23]. The median follow-up time in this study was limited though with 2,6 years, compared to 4,9 years in our study, resulting in limited follow-up and only five-year survival rates. More importantly the results of our study, with a smaller population and shorter follow-up, were in concordance with another large series of 402 osteosarcoma patients and 11,3 years of median follow-up. In this study, a plateau in events (local recurrence, new or progressive distant metastasis or death) was seen five years after treatment [24].

Interestingly, the survival curve for chordoma appeared to be clearly different with elongated median time to local recurrence and distant metastasis along with a steady decline in survival during five-year follow-up. This was resembled by the incidence of local recurrence and metastasis. According to literature, stabilisation in disease specific survival is seen after 15 years. Furthermore, older age above 59 years (accompanied by comorbidities) was identified as a prognostic factor for worse survival [25]. Therefore, although chordoma is also defined a high-grade bone sarcoma, a different follow-up strategy seems indicated. Extended follow-up after five years without prolonged intervals is deemed to be justified for this entity (Table 5, Table 6).

**Table 5:** Included subtypes based on ICD-O-3 coding and grade.

Sarcoma subtype (WHO 2013)	Morphology code
*High-grade (2/3) chondrosarcoma*	
Chrondrosarcoma NOS	9220/3 + 9231/3
Dedifferentiated chondrosarcoma	9243/3
*Surface osteosarcoma*	
Parosteal osteosarcoma	9192/3
Periosteal osteosarcoma	9193/3
High-grade surface osteosarcoma	9194/3
*High-grade osteosarcoma*	
Osteosarcoma NOS	9180/3
Chondroblastic osteosarcoma	9181/3
Fibroblastic osteosarcoma	9182/3
Teleangiectatic osteosarcoma	9183/3
Osteosarcoma in Paget’s disease	9184/3
Small-cell osteosarcoma	9185/3
Central osteosarcoma	9186/3
Intracortical osteosarcoma	9195/3
*Ewing sarcoma*	9260/3
*Angiosarcoma of bone*	
Epithelioid hemangioendothelioma NOS	9133/3
Hemangiosarcoma	9120/3
*Sarcoma of bone NOS*	
Sarcoma	8800/3
Splindle cell sarcoma	8801/3
Small-cell sarcoma	8803/3
Chordoma	9370
Dediferentiated Chordroma	9372
Adamantinoma	9261

Grade: 3 = high.

**Table 6:** Excluded subtypes based on ICD-O-3 coding and grade.

Sarcoma subtype (WHO 2013)	Morphology code
Giant cell sarcoma	8802
Epithelioid sarcoma	8804
Fibrous histiocytoma	8830
Leomyoma / Leomyosarcoma	8890
Fibrous mesothelioma	9051
Chondroblastoma	9230
Giant cell tumour of bone	9250
Ameloblastoma	9310
Clear cell odontogenic carcinoma	9341
Chondroid chordoma	9371
Atypical Cartilaginous Tumour	9220 / Low-grade
Low-grade central osteosarcoma	9186 / Low-grade
Clear-cell chondrosarcoma	9242 / Low-grade
Periosteal chondrosarcoma	9221 / Low-grade

Overall survival rates for all entities in our study were lower in comparison with available literature [4], [6], [7], [8], [24], [26], [27]. This could be explained by inclusion of patients with primary metastatic disease and the exclusion of patients with missing follow-up data which likely resulted in selection bias. Furthermore, based on our database, the cause of death could not be linked to the disease itself or other causes (e.g. after adjuvant treatment, other disease, unknown). This likely affected overall survival negatively as well.

Follow-up intervals were not recorded in our database and therefore not available for analysis. Treatment and follow-up of bone sarcoma patients is centralised in four hospitals, generic follow-up intervals in the Netherlands have been described earlier [28]. At present, follow-up intervals for adults with high-grade chondrosarcoma, high-grade osteosarcoma and Ewing sarcoma are every 3 months in the first year after treatment, every 4 months in the second year and then prolonged towards a 6-month-interval until 5 years of follow-up. A 1-year-interval is used until 10 years of follow-up. This surveillance scheme is consistent with well-established international guidelines [29], [30], [31]. Puri et al. found that a less-intensive follow-up protocol did not result in a decreased overall survival. Comparison is difficult however due to the heterogeneous study population with both soft-tissue and bone sarcoma of the limb included [18].

Based on the findings and sample size of our study, a new recommendation with specified follow-up intervals for high-grade bone sarcomas would not be justified. However, our findings regarding time to local recurrence and distant metastasis questions the necessity for extended surveillance after five years of follow-up for high-grade osteosarcoma and Ewing sarcoma, consistent with the existing literature [10], [23].

Limitations of our study are the retrospective study design. However, the registration of the pathological data from PALGA is a continuous prospective process which amplifies our results. Although complete follow-up was essential for our analysis, this resulted in a relatively small sample size and short follow-up time for high-grade chondrosarcoma, high-grade osteosarcoma and Ewing sarcoma which could have resulted in selection bias. Moreover, detection of local recurrences and distant metastasis in our study is based on the PALGA database for which pathological samples from biopsy or resection are mandatory. Since our inclusion is based on pathological data, we may have missed cases where no histological sampling was performed.

For future research, larger cohorts could be valuable to validate the findings in our study. In addition, we believe that cost-effectiveness analyses of follow-up surveillance programmes are important. Future research must address the psychological distress of follow-up as well and try to find a healthy balance between usefulness and distress. Finally, a prediction model for clinical guidance to facilitate individualized follow-up would be the next step for high-grade bone sarcoma patients similar to the PERSARC model for soft-tissue sarcoma [32].

In conclusion, our study shows a plateau in new local recurrences and distant metastatic events four years after initial treatment for patients with high-grade osteosarcoma and Ewing sarcoma. Even though our study is based on a nationwide population, collaborative research with larger groups is needed in order to do provide a solid scientific basis for future recommendations for follow-up interval and duration in the heterogenous patient population with bone sarcoma. Importantly, with the data presented here, we believe that the discussion regarding the purpose of extended follow-up and its value for the individual patient initially treated with curative intend should be intensified.

## CRediT authorship contribution statement

**Louren M. Goedhart:** Conceptualization, Formal analysis, Project administration, Writing – original draft. **Vincent K.Y. Ho:** Conceptualization, Data curation, Formal analysis, Writing – review & editing. **Joris J.W. Ploegmakers:** Supervision, Writing – review & editing. **Ingrid C.M. van der Geest:** Writing – review & editing. **Michiel A.J. van de Sande:** Writing – review & editing. **Jos A. Bramer:** Writing – review & editing. **Martin Stevens:** Methodology, Supervision, Writing – review & editing. **Paul C. Jutte:** Conceptualization, Supervision, Writing – review & editing.

## Declaration of Competing Interest

The authors declare that they have no known competing financial interests or personal relationships that could have appeared to influence the work reported in this paper.

## Appendix A

Classification of sarcoma of bone based on the International Classification of Diseases for Oncology (ICD-O-3) and the World Health Organization (WHO) classification 2013 combined with clinicopathological expertise.
